# Downregulation of miR-26b-5p, miR-204-5p, and miR-497-3p Expression Facilitates Exercise-Induced Physiological Cardiac Hypertrophy by Augmenting Autophagy in Rats

**DOI:** 10.3389/fgene.2020.00078

**Published:** 2020-02-19

**Authors:** Jie Qi, Xue Luo, Zhichao Ma, Bo Zhang, Shuyan Li, Jun Zhang

**Affiliations:** ^1^ College of Physical Education, Shanghai Normal University, Shanghai, China; ^2^ Medical College, Yangzhou Polytechnic College, Yangzhou, China; ^3^ The School of Physical Education, Wuhan Business University, Wuhan, China; ^4^ College of Physical Education, Yangzhou University, Yangzhou, China

**Keywords:** autophagy, exercise, insulin-like growth factor 1, cardiac hypertrophy, microRNA

## Abstract

Exercise-induced autophagy is associated with physiological left ventricular hypertrophy (LVH), and a growing body of evidence suggests that microRNAs (miRNAs) can regulate autophagy-related genes. However, the precise role of miRNAs in exercise induced autophagy in physiological LVH has not been fully defined. In this study, we investigated the microRNA–autophagy axis in physiological LVH and deciphered the underlying mechanism using a rat swimming exercise model. Rats were assigned to sedentary control (CON) and swimming exercise (EX) groups; those in the latter group completed a 10-week swimming exercise without any load. For *in vitro* studies, H9C2 cardiomyocyte cell line was stimulated with IGF-1 for hypertrophy. We found a significant increase in autophagy activity in the hearts of rats with exercise-induced physiological hypertrophy, and miRNAs showed a high score in the pathway enriched in autophagy. Moreover, the expression levels of miR-26b-5p, miR-204-5p, and miR-497-3p showed an obvious increase in rat hearts. Adenovirus-mediated overexpression of miR-26b-5p, miR-204-5p, and miR-497-3p markedly attenuated IGF-1-induced hypertrophy in H9C2 cells by suppressing autophagy. Furthermore, miR-26b-5p, miR-204-5p, and miR-497-3p attenuated autophagy in H9C2 cells through targeting ULK1, LC3B, and Beclin 1, respectively. Taken together, our results demonstrate that swimming exercise induced physiological LVH, at least in part, by modulating the microRNA–autophagy axis, and that miR-26b-5p, miR-204-5p, and miR-497-3p may help distinguish physiological and pathological LVH.

## Introduction

The two types of left ventricular hypertrophy (LVH), namely physiological and pathological LVH, differ greatly in the left ventricular phenotype. Both of them have an increased myocyte volume and heart size. The difference is that physiological LVH is induced by aerobic exercise training, postnatal growth, and pregnancy, and characterized by unchanged fetal and apoptosis gene expression and increased cardiac function while pathological LVH is stimulated by pressure or volume overload or cardiomyopathy, and characterized by apoptosis and fibrosis and depressed cardiac function (Bernardo et al., 2010; Nakamura and Sadoshima, 2018; Oldfield et al., 2019). For example, LVH induced by swimming exercise training is an adaption for a chronic increase in hemodynamic overload (Xiao et al., 2014; Bernardo et al., 2018), whereas myocardial infarction induced pathological LVH is associated with increased fibrosis, lowered aerobic capacity, and maladaptive remodeling (McMullen and Izumo, 2006; Dorn, 2007; Schiattarella and Hill, 2015). The physiological LVH exerts cardioprotection in patients with cardiovascular diseases. However, the mechanism of exercise-induced LVH remains unclear.

Physical exercise has been identified as an inducer of autophagy (Halling and Pilegaard, 2017; Martin-Rincon et al., 2018). Exercise was reported to induce autophagy in several organs such as cardiac tissue, skeletal muscle, liver, pancreas, hippocampus, and adipose tissue (Brandt et al., 2018; Li et al., 2018b). Induction of skeletal and cardiac muscle autophagy during endurance training triggers beneficial adaptive changes in mitochondrial metabolism and is associated with enhanced physical fitness (Lira et al., 2013). Autophagy is required for exercise training-induced skeletal muscle adaption and for the improvement of physical performance (Gottlieb and Mentzer, 2013; Fritzen et al., 2016; Sanchez, 2016). However, the mechanism of exercise-induced autophagy remains unknown.

Accumulated evidences showed that microRNA (miRNA, miR) networks changed in response to exercise contributed to physiological cardiac hypertrophy (Carè et al., 2007; Fernandes et al., 2011; Fernandes et al., 2015). However, different types of exercise training have been reported to cause changes in different miRNAs (Martinelli et al., 2014; Melo et al., 2015; Ramasamy et al., 2015). MiRNAs could target autophagy-related genes and negatively regulate their activities (Shen et al., 2016; Aredia and Scovassi, 2017; Chen et al., 2017). MiRNAs modulate autophagy at different stages, such as at autophagic induction, vesicle nucleation, and vesicle elongation and completion stages, by targeting autophagy-related genes or autophagy complexes (Martinelli et al., 2014; Zhang and Chen, 2018). Although a growing body of evidence indicates that miRNAs regulate autophagy-related genes, their precise role in autophagy pathways has not been fully defined in physiological cardiac hypertrophy. Therefore, we established physiological *in vitro* and *in vivo* LVH models to investigate the microRNA–autophagy axis in physiological hypertrophy.

## Materials and Methods

### Animal Care and Exercise Protocols

All care policies and procedures in this study conformed to the Guide for the Care and Use of Laboratory Animals published by the US National Institutes of Health (NIH publication No. 85–23, revised 1996) and were approved by the Ethics Committee for the Use of Experimental Animals at Shanghai Normal University, China. Female Wistar rats (200 ± 20 g, n = 32) were fed a standard diet, exposed to a 12-h light–12-h dark cycle, and maintained in a constant room temperature (22 ± 2°C) and humidity (50 ± 10%) (Fernandes et al., 2011). The rats were randomly assigned to two groups: 1) sedentary control (CON, n = 16) and 2) swimming exercise (EX, n = 16). For 10 weeks, from Monday to Friday, the rats in the EX group completed a 1-h swimming exercise schedule without any load. The exercise training was performed by placing the rats in a swimming pool (150 cm × 60 cm × 70 cm) filled with warm water to a depth of 60 cm. The pool was divided by plastic barriers into eight lanes. The water temperature was maintained at 31 ± 1°C. All the animals were weighed once a week. In contrast, rats in the CON group were exposed to the water twice weekly—they were placed in the swimming pool at these junctures for 10-min sessions. The O_2_ uptake for rats swimming individually was about 50–65% of the maximum oxygen uptake. This low-intensity, long-period swimming exercise protocol is effective for promoting cardiovascular adaptations and for increasing muscle oxidative capacity. These protocols were previously reported by Fernandes et al. (2011) and Oliveira et al. (2009).

### Measurement of Blood Pressure and Heart Rate

Blood pressure (BP) and heart rate (HR) were measured after 24 h of the last exercise session. The hemodynamic parameters of rats were measured with a blood pressure analyzer (BP-98A; Softron, Tokyo, Japan), after they had been placed undisturbed in a restrainer for a minimum of 5 min, following the tail-cuff method. The recorded data indicated the average of all values of systolic blood pressure (SBP), diastolic blood pressure (DBP), HR, and mean arterial pressure (MBP) over the entire recording time of 20 min.

### Measurement of Cardiac Hypertrophy

The rats were euthanized by cervical dislocation under anesthesia induced by intraperitoneal injection of 3% sodium pentobarbital. To measure the cardiac function, the hearts were stopped at diastole by perfusion of 14 mM KCl. After the heart weight (HW) was measured, the left ventricle (LV) was dissected corresponding to the remaining tissue upon the removal of both atria and the free wall of the right ventricle (RV). The interventricular septum remained as part of the LV. Left cardiac hypertrophy was assessed by determining the ratio of LV weight to HW (HW/BW) (Fernandes et al., 2011). Then the LVs were fixed with 10% formalin and embedded in paraffin. Heart sections (5 μm in thickness) were made and stained with hematoxylin and eosin (HE) for imaging the heart structures. Four random sections from each heart were visualized using light microscopy at 40X magnification. Myocytes with a visible nucleus and intact cellular membrane were chosen for determination of the myocyte diameter. The width of individually isolated cardiomyocytes were displayed on a viewing screen that was manually traced, across the middle of the nuclei, with a digitizing pad and determined using a computer-assisted image analysis system (ScopePhoto 3.0 for Scope Technology). For each group, 20 visual fields were assayed.

### Transmission Electron Microscopy

Transmission electron microscopy was performed by the method described by Nadal and Gold (2012). In brief, freshly prepared cardiac tissues were fixed overnight in 2% glutaraldehyde at 4°C. Thereafter, the sections were immersed in 1% buffered osmium tetroxide for 2 h. The specimens were then dehydrated through a graded series of ethanol and embedded in an epoxy resin. The specimens were then sliced into ultrathin sections (80 nm) with 0.1% citrate lead and 10% uranium acetate. The sections were examined under a transmission electron microscope (Hitachi, Tokyo, Japan).

### Cell Culture

The rat H9C2 cells were purchased from the cell bank of the Chinese Academy of Sciences (Shanghai, China) and cultured in Dulbecco's modified Eagle's medium (Gibco, USA), supplemented with 100 U/ml penicillin–streptomycin, and 10% fetal bovine serum (BSA) in a 5% CO_2_ humidified atmosphere at 37°C. The cells were grown at a density of 4 × 10^5^ cells/ml. For the hypertrophy model, cells were grown with 10 μM insulin-like growth factor (IGF-1, Sigma-Aldrich, MO, USA) for 48 h at 37°C in a 5% CO_2_ incubator.

### Immunofluorescence Staining

For immunofluorescence analysis, H9C2 cells were fixed with 4% paraformaldehyde for 15 min, permeabilized with 0.1% Triton X-100 in PBS for 10 min, and blocked with 3% BSA solution for 1 h. These cells or paraffin-embedded sections were incubated overnight with a microtubule-associated protein 1 light chain 3B (LC3B) antibody (Cell Signaling Technology; 1:200) at 4°C, washed, and stained with a fluorescent dye (Alexa Fluor 555)-conjugated secondary antibody (Cell Signaling Technology; 1:200). The tissue sections or cells were subsequently mounted with a fluorescent mounting medium (Beyotime Biotechnology, Shanghai, China) and coverslips were placed over them. Immunofluorescence was analyzed with a fluorescence microscope (Carl Zeiss, Germany), and the number of LC3 puncta was determined using Image-Pro Plus 6.0 software.

### Ribonucleic Acid Extraction and Microribonucleic Acid Microarray

Total RNA and miRNAs were extracted using TRIzol (Invitrogen, Waltham, MA) and miRNeasy mini kit (QIAGEN, Germany), respectively, according to the manufacturer's instructions. After quantitating the RNA with NanoDrop 1000 spectrophotometer (NanoDrop Technologies, USA) and standard denaturing agarose gel electrophoresis, samples from two animals in each group were pooled and labeled using the miRCURY™ Hy3™/Hy5™ Power Labeling Kit (Exiqon, Vedbaek, Denmark). They were then hybridized on the miRCURY™ LNA Array (v.16.0) (Exiqon, Vedbaek, Denmark). Next, the slides were scanned using the Axon GenePix 4000B Microarray Scanner (Axon Instruments, Foster City, CA). The scanned images were then imported into GenePix Pro 6.0 software (Axon) for grid alignment and data extraction. Replicated miRNAs were averaged and miRNAs that intensities ≥ 30 in all samples were chosen for calculating normalization factor. Expressed data were normalized using the Median normalization. After normalization, significant differentially expressed miRNAs between two groups were identified through Fold change (≥1.5) and P-value (P ≤ 0.05). An electronic link to the miRNA microarray platform is available at http://www.ncbi.nlm.nih.gov/geo/query/acc.cgi?acc=GPL11434.

### Bioinformatics

After microarray analyze, predicted target genes of candidate miRNAs were determined using three bioinformatics prediction tools: miRBase (http://www.mirbase.org/), TargetScan (http://www.targetscan.org, Jacobsen et al., 2013), and miRDB (http://www.mirdb.org/miRDB/, Liu and Wang, 2019). MiRBase was used to define the miRNA sequences, and TargetScan and miRDB were used to predict the target genes of miRNAs. The predicted miRNA target genes were then subjected to Gene Ontology (GO) and Kyoto Encyclopedia of Genes and Genomes (KEGG) analyses using DAVID (http://david.abcc.ncifcrf.gov/) online (Han et al., 2012). Eighty-three pathways were enriched with the miRNA target genes and the autophagy pathway was the second pathway of the top 10. The autophagy pathway included 16 miRNAs. Among them, one most upregulated (fold change >2.5) and four most downregulated (fold change <0.25) miRNA were selected and homology was analyzed. Again, TargetScan and miRDB were used to predict the target genes of the selected miRNAs, and the information from the two databases were integratively considered.

### Quantitative Real-Time Polymerase Chain Reaction Analysis of Messenger Ribonucleic Acid and Microribonucleic Acid Expression

For reverse transcription, total RNA was prepared from cells using the TRIzol reagent according to the manufacturer's instructions (Invitrogen). Total RNA (2 µg) was reverse transcribed using the PrimeScript™ RT Master Mix Kit (Takara, Kusatsu, Japan) and Mir-X™ miRNA First-Strand Synthesis Kit (Clontech, USA) for SYBR Green PCR, respectively. Quantitative real-time PCR (qRT-PCR) was performed in triplicate using the ABI 7500 System (ABI, New York, USA) in a 20-μl reaction volume. The real-time PCR and data collection were subsequently performed, as described previously (Ma et al., 2013 **). The relative expression levels of the indicated mRNAs normalized against glyceraldehyde 3-phosphate dehydrogenase (GAPDH) mRNA were calculated using the 2^−ΔΔCT^ method. The primer sequences used for RT-qPCR are listed in **Supplementary Table 1**. For microRNAs, the expression level of U6 was used as an internal control.

### Protein Extraction and Western Blot Analysis

Western blotting was performed following the standard method. Briefly, the samples were placed in protein extraction solution (RIPA) and ultrasonicated at maximum speed at 4°C for 30 s (Sonics, Newtown, USA). The homogenate was centrifuged at 12,000 × *g* at 4°C for 30 min. After denaturation, the samples were subjected to 10% sodium dodecyl sulfate polyacrylamide gel electrophoresis (SDS-PAGE) and the resolved proteins were transferred onto polyvinylidene fluoride membranes (Millipore, Billerica, USA). The membranes were blocked for 1 h with 5% milk and then probed with anti-LC3B (cat. no. 3868; 1:1,000), anti-Beclin 1 (cat. no. 3495; 1:1,000), anti-unc-51 like autophagy activating kinase 1 (ULK1) (cat. no. 8054; 1:1,000), anti-Atg4B (cat. no. 5299; 1:1,000), anti-Atg5 (cat. no. 12994; 1:1,000), anti-Atg7 (cat. no. 2631; 1:1,000), anti-Atg12 (cat. no. 4180; 1:1,000), anti-ANP (cat. no. 209232, 1:1,000), anti-brain natriuretic peptide (BNP) (cat. no. 19645, 1:1,000), or anti-SQSTM1 (cat. no. 39749; 1:1,000) antibodies, which were all purchased from Cell Signaling Technology, MA, USA, at 4°C overnight. After washing three times with TBST (Thermo Fisher Scientific, Inc., Waltham, MA), the membranes were incubated with the corresponding horseradish peroxidase-conjugated secondary antibodies (goat anti-rabbit; cat. no. ab6721; 1:2,000; Cell Signaling Technology) for 2 h at room temperature. The immunoreactive protein bands were visualized with Pierce ECL Plus Western Blot Substrate (Thermo Fisher Scientific, Rockford, IL, USA). GAPDH was used as an internal loading control. The band intensity was quantified using ImageJ software (NIH) and was defined as fold-change relative to the band intensity in the CON samples after normalization against GAPDH.

### Adenovirus-Mediated Microribonucleic Acid Infection

We performed adenovirus-mediated infection of H9C2 cells for overexpression of miR-26b-5p (Ad-26b-5p), miR-204-5p (Ad-204-5p), miR-497-3p (Ad-497-3p), let-7a-5p (Ad-7a-5p), and miR-181a-5p sponges (Ad-181a-5p) (Vigenebio, Shandong, China) at a multiplicity of infection 10, respectively. After 48 h of infection, the cells were collected for RT-qPCR and western blot assays.

### Statistical Analysis

Data were presented at mean ± SD. Statistical analysis was performed using Prism Software (GraphPad Prism 5.0). For analysis of two groups, Student's t-test was used; for comparison of three or more groups, one-way ANOVA followed by Bonferroni's post-test was applied. For each analysis, P < 0.05 was considered significant.

## Results

### Ten-Weeks Swimming Exercise Induces Physiological Cardiac Hypertrophy in Rats

**Figure 1:**
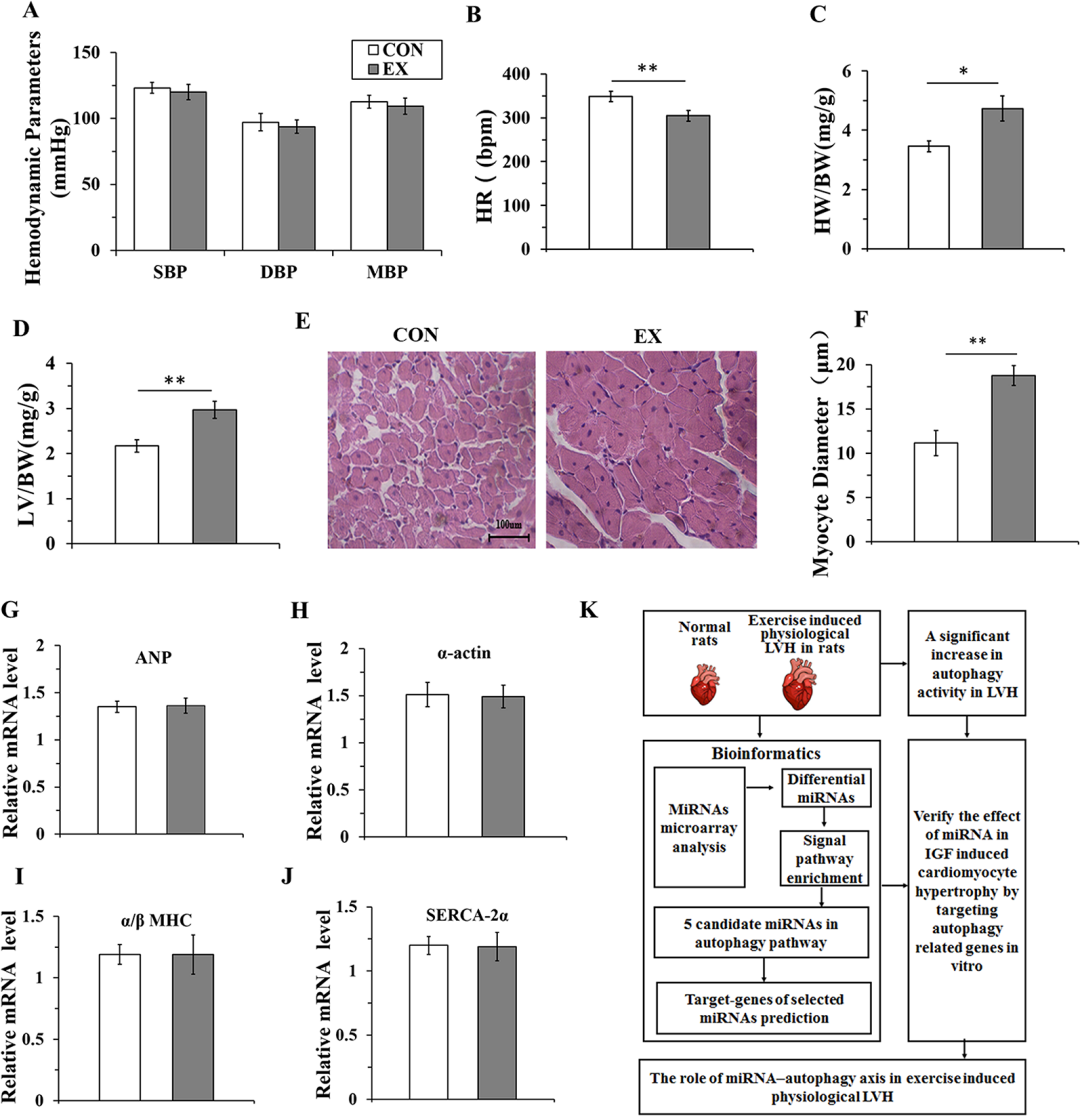
Ten-weeks swimming exercise induces physiological cardiac hypertrophy in rats. **(A)**, The systolic blood pressure (SBP), diastolic blood pressure (DBP), and mean arterial pressure (MBP) in the control (CON) and swimming exercise (EX) groups. **(B)**, The heart rate (HR) in the EX group. **(C**, **D)**, Ten-week swimming exercise increased the HW/BW and LV/BW ratios in the EX group. BW, body weight; LV, left ventricular weight; HW, heart weight. **(E**, **F)**, Hematoxylin staining of LV myocytes in the CON group and EX group. Images were obtained at a magnification of ×400. **(G–J)**, The levels of atrial natriuretic polypeptide (ANP) and the skeletal muscle α-actin (α-actin), and the ratio of α/β-myosin heavy chain (α/β-MHC) and levels of sarco-endoplasmic reticulum Ca^2+^-ATPase (SERCA-2α) in the CON group and EX group. **(K)**, Diagram depicting the experimental process of this study. * P < 0.05, ** P < 0.01, EX group (n=16) *vs*. CON group (n=16). Data are presented as mean ± SD. Statistical significance was evaluated with the two-tailed Student's t test.

To evaluate whether 10-week swimming exercise induced LVH, the systolic, diastolic, and mean blood pressure, and heart rate were measured for rats in the CON (n=16) and EX groups (n=16) (**Figures 1A, B**). There were no differences in the blood pressure between the two groups (P > 0.05), but the heart rate in the EX group was significantly lower than in the CON group (304.6 ± 12.1 bpm *vs*. 348.8 ± 11.7 bpm; P < 0.05) after 10-week swimming exercise. The LV/BW and HW/BW ratios were used to evaluate LVH. Compared with the CON group, the HW/BW ratio was markedly increased in the EX group (4.73 ± 0.42 *vs*. 2.17 ± 0.14 for EX *vs*. CON; P < 0.05, n = 16; **Figure 1C**). The value of LV/BW in the EX group was 1.37-fold (2.97 ± 0.19; P < 0.01, **Figure 1D**), which was also higher than in the CON group (2.17 ± 0.14). Moreover, as evident from the HE staining (**Figure 1E**), in the CON group, the myocardial cells were arranged orderly and there was less amount of extracellular matrix, whereas in the EX group, the myocardial fibers were evenly colored, the myocardial cells were arranged more orderly, the number of nuclei was increased, and the structure was normal. Furthermore, when compared with the CON group, a significant increase in the diameter of the LV myocytes was observed in the EX group (14.77 ± 1.64 *vs*. 12.15 ± 1.42 μm for EX *vs*. CON; P < 0.05, n = 16; **Figure 1F**).

The indices of pathological cardiac hypertrophy (Lowes et al., 1997; Weinberg et al., 1999), such as the atrial natriuretic polypeptide (ANP), sarco-endoplasmic reticulum Ca^2+^-ATPase (SERCA-2α), the skeletal muscle α-actin, and the ratio of α/β-myosin heavy chain (α/β-MHC), were not altered in the EX group compared to those in the CON group (**Figures 1G–J**). Diagram depicting the experimental process of this study was shown in **Figure 1K**.

### Autophagy Is Markedly Enhanced in Swimming-Induced Physiological Cardiac Hypertrophy in Rats

**Figure 2:**
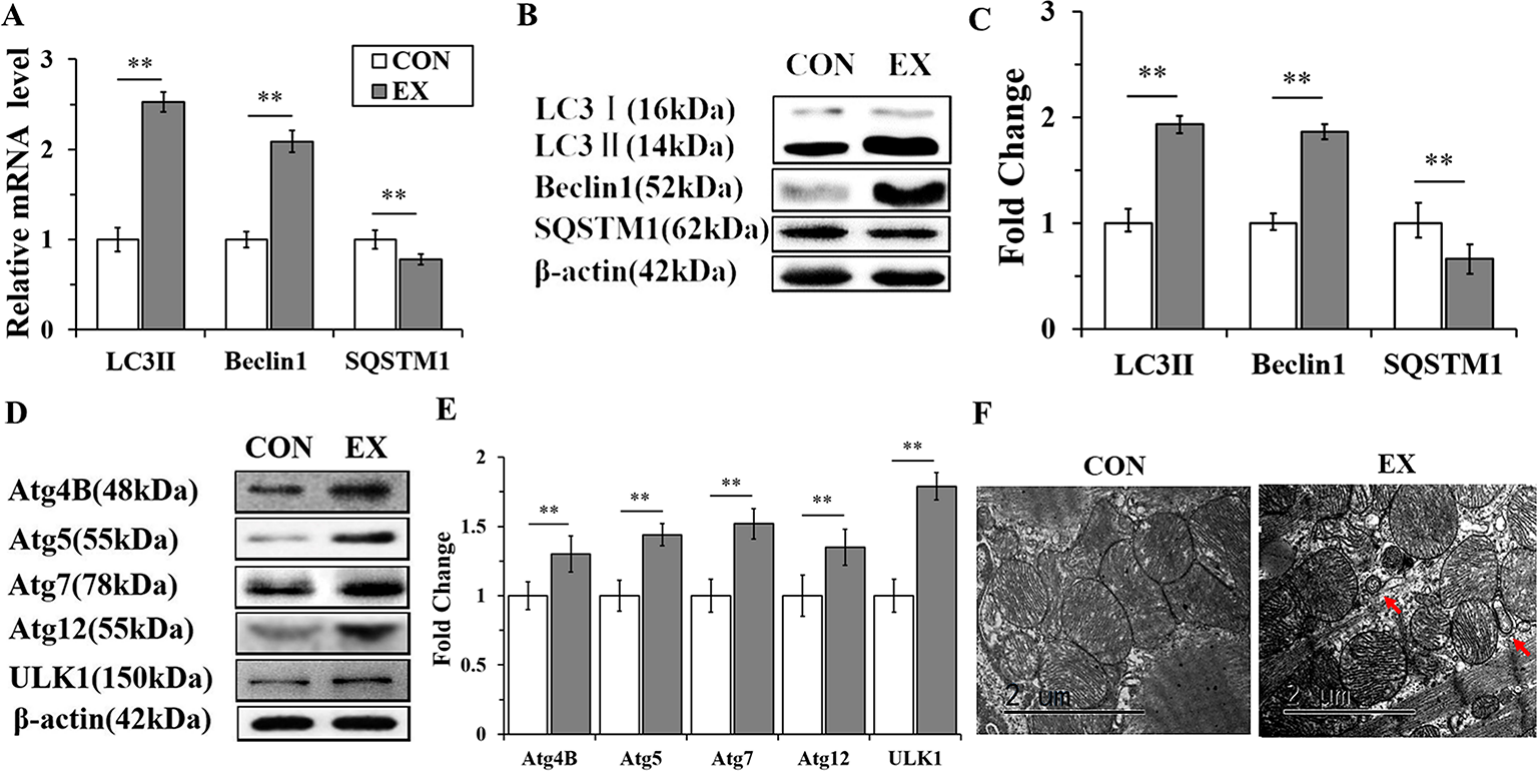
Autophagy is markedly enhanced in swimming-induced physiological cardiac hypertrophy in rats. **(A)**, Real-time quantitative (RT-q)PCR analysis for mRNA expression of LC3B, Beclin 1, and SQSTM1 (relative to β-actin). **(B**, **C)**, Western blot analysis for LC3B, Beclin 1, and SQSTM1 (relative to β-actin). **(D**, **E)**, Western blot analysis of samples from CON and EX group detected Atg4B, Atg5, Atg7, Atg12 and ULK1. **(F)**, Representative transmission electron microscopic images of autophagosomes. Autophagosomes (red arrows). Images were obtained at a magnification ×13,000. Scale bar, 2 μm. ** P < 0.01, EX group (n=16) *vs*. CON group (n=16). Data are presented as means ± SD. Statistical significance was evaluated with the two-tailed Student's t test.

To detect the activation of autophagy, the expression levels of LC3, Beclin 1, and SQSTM1 mRNAs and proteins were assessed by RT-qPCR and western blot analyses, respectively. The mRNA levels of LC3 II and Beclin 1 in the EX group (n=16) were obviously increased, by 2.53± 0.15- (P < 0.01) and 2.09 ± 0.13-fold (P < 0.01), respectively, compared to the respective levels in the CON group (n = 16), whereas the level of SQSTM1 mRNA was significantly decreased (P < 0.01) (**Figure 2A**). Furthermore, the expression levels of LC3 II and Beclin 1 proteins were upregulated in the EX group (the increase was by 1.93 ± 0.17- and 1.86 ± 0.12-fold compared with the respective levels in the CON group, and in both cases, the increase was significant at P < 0.01); the SQSTM1 protein level showed an obvious decrease with respect to its level in the CON group; P < 0.01 (**Figures 2B, C**).To further confirm the swimming exercise induced autophagy, the protein levels of Atg4B, Atg5, Atg7, Atg12, and ULK1 in the LV were determined by western blot analysis. As shown in **Figures 2D, E**, the expression levels of these five proteins were significantly increased by 1.3 ± 0.18- (P < 0.05), 1.44 ± 0.12- (P < 0.01), 1.52 ± 0.16- (P < 0.01), 1.35 ± 0.11- (P < 0.01), and 1.21 ± 0.1- (P < 0.05) fold, respectively, in the EX group compared to their levels in the CON group. In addition, the ultrastructure of rat hearts were observed by electron microscopy. The double membrane structure of autophagosomes was observed in the EX group, whereas in the CON group, we did not observe the typical structure of autophagosomes (**Figure 2F**).

### Microribonucleic Acid Targeting the Autophagy Pathway Are Significantly Downregulated in Physiological Cardiac Hypertrophy

**Figure 3:**
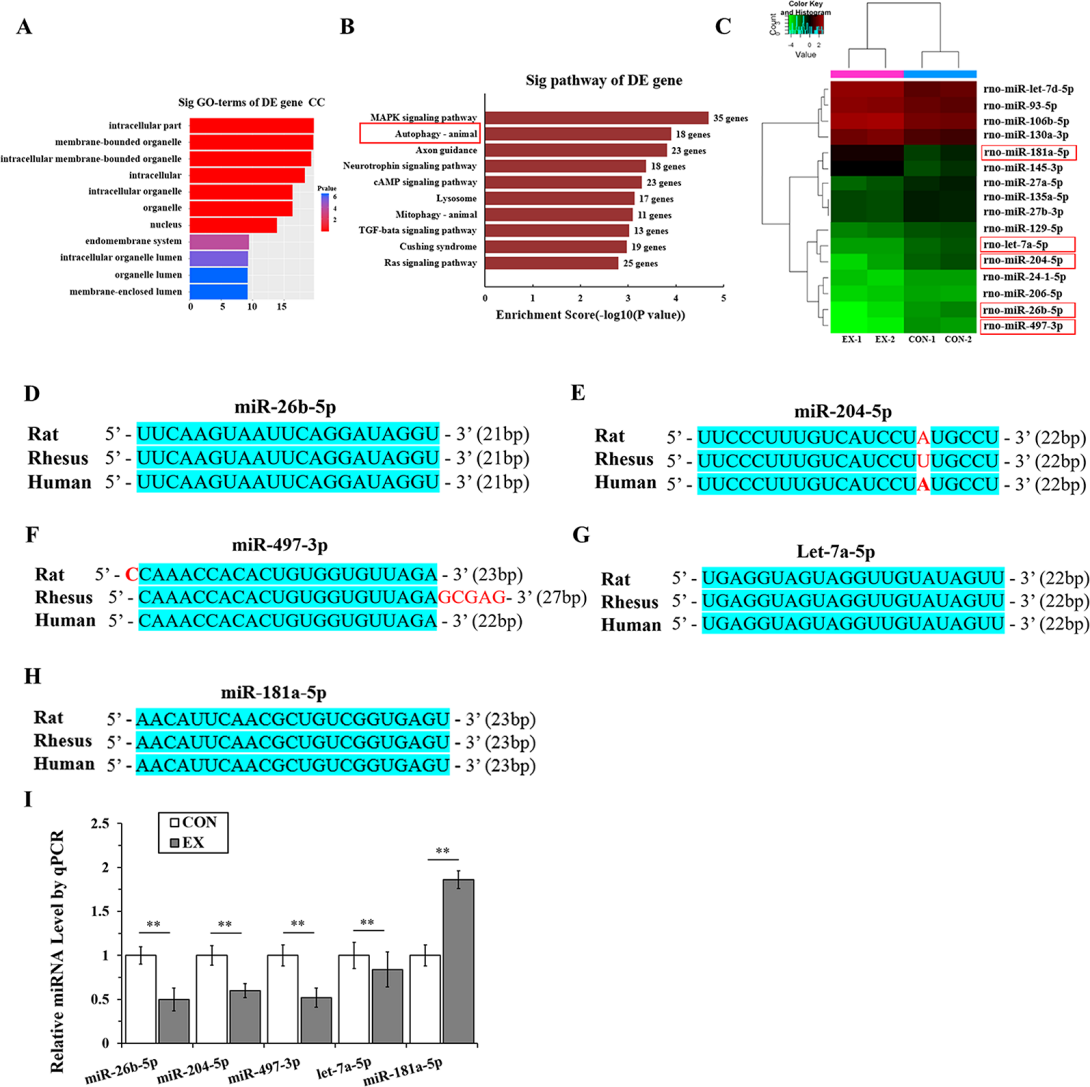
MicroRNAs (miRNAs) targeting the autophagy pathway are significantly downregulated in physiological cardiac hypertrophy. **(A),** Gene ontology (GO) analyses showed that most of the miRNA target genes were enriched in cell membrane structure. **(B)**, Kyoto Encyclopedia of Genes and Genomes (KEGG) analyses. Horizontal axis indicates pathway enrichment score; vertical axis indicates the pathway name. The data label on the right stands for the differentially expressed (DE) gene number associated with the pathway. **(C)**, Heat map for clustering analysis of miRNA expression data in the CON (n = 2) and EX (n = 2) groups; the miRNAs in the red box are those that bind to the autophagy-related genes in LVH. **(D–H)**, The sequence homology among rat, rhesus, and human miRNAs; the blue shading area represents the same sequence. **(I)**, Determination of miRNAs by real-time (RT)-qPCR. Targeted miRNAs were normalized with respect to the U6 levels. ** P < 0.01, EX group (n=16) *vs*. CON group (n=16). Data are presented as means ± SD. Statistical significance was evaluated with the two-tailed Student's t test.

Through miRNA microarray analysis, we found 216 differential miRNA (77 upregulated, 139 downregulated) between normal and exercised heart. GO analyses showed that most of the miRNA target gene was enriched in cell membrane structure (**Figure 3A**). A total of 83 pathways were enriched and we listed the top 10 pathways in the decreasing order of their enrichment scores. The autophagy pathway was the second most enriched pathway (**Figure 3B**, **Supplementary Table 2**). The autophagy pathway included 16 miRNAs (**Figure 3C**). Among them, one most upregulated (fold change >2.5) and four most downregulated (fold change <0.25) miRNA were selected and homology was analyzed. After analyzing the gene homology among rat, rhesus, and human sequences (**Figures 3D–H**) using DNAMAN software, we found that miR-26b-5p, miR-204-5p, miR-497-3p, let-7a-5p, and miR-181a-5p in rat were highly homologous to their human counterparts. We further analyzed these five miRNAs using RT-qPCR and found that miR-26b-5p, let-7a-5p, miR-204-5p, and miR-497-3p were significantly downregulated in the EX group (P < 0.01), whereas miR181a-5p was upregulated in the EX group (1.86-fold increase over expression in the CON group; P < 0.05) (**Figure 3I**).

### Overexpression of miR-26b-5p, miR-204-5p, and miR-497-3p Attenuates IGF-1 Induced Cardiomyocyte Hypertrophy by Suppressing Autophagy

**Figure 4:**
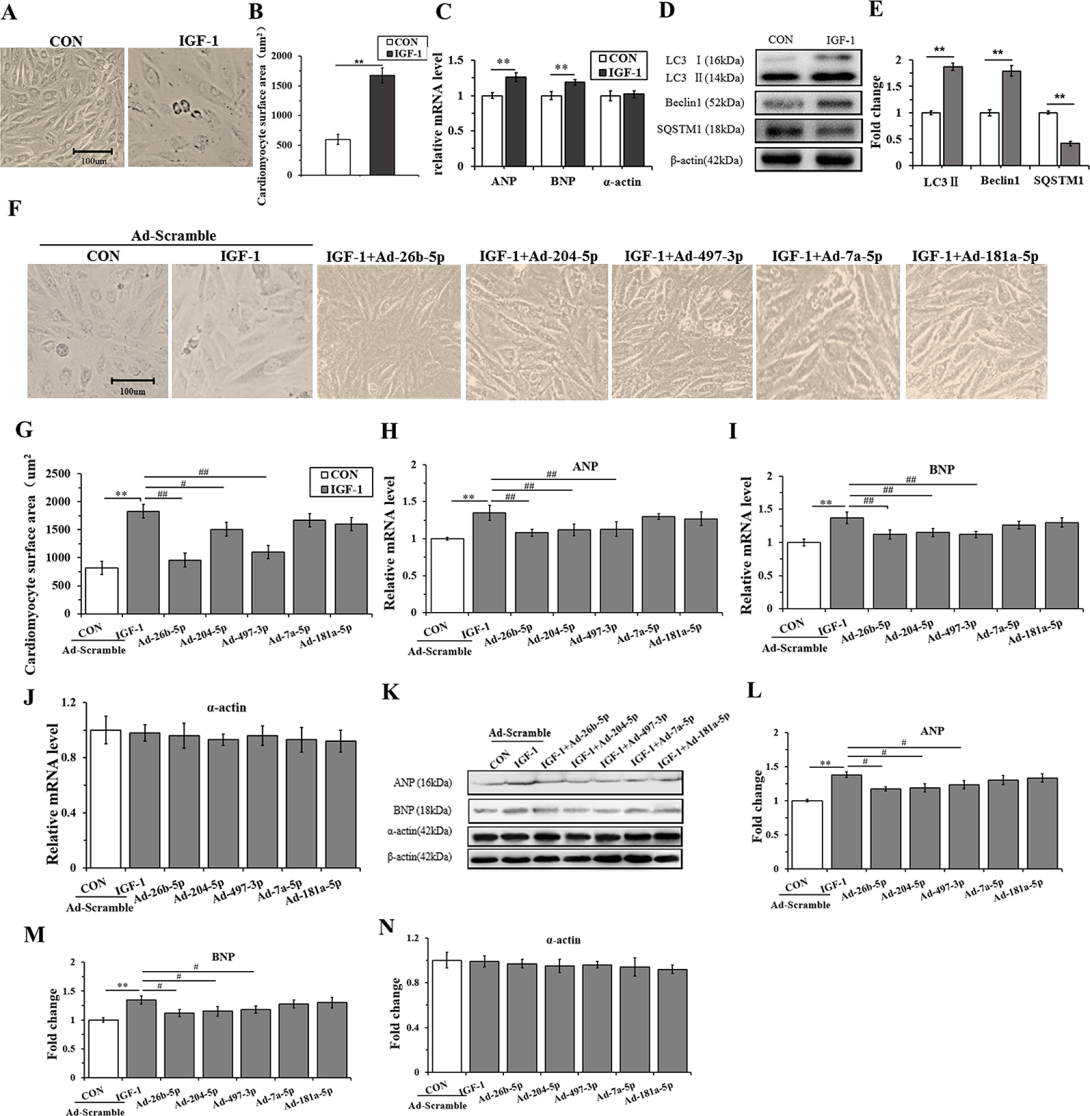
Overexpression of miR-26b-5p, miR-204-5p, and miR-497-3p attenuates IGF-1 induced cardiomyocyte hypertrophy. **(A, B)**, The morphology of H9C2 cells treated with IGF-1 (10 µM)for 48 h. **(C)**, The messenger RNA (mRNA) levels of atrial natriuretic polypeptide (ANP), brain natriuretic peptide (BNP), and α-actin in H9C2 cells treated with IGF-1 (10 µM). **(D, E)**, The protein expression of autophagy maker (LC3B, Beclin1, and SQSTM1) of H9C2 cells treated with IGF-1 (10 µM). **(F, G)**, Cardiomyocyte surface area of H9C2 cells in response to IGF-1 (10 µM) with adenovirus-mediated microRNA (miRNA) infection. **(H–J)** mRNA levels of cardiomyocyte hypertrophy makers of H9C2 cells treated with IGF-1 (10 µM) and adenovirus mediated miRNAs. **(K–N)** Protein levels of cardiomyocyte hypertrophy makers of H9C2 cells treated with IGF-1 (10 µM) and adenovirus mediated miRNAs. ** P < 0.01, IGF+scramble (n=6) *vs*. CON+scramble group (n=6). ^#^P < 0.05, ^##^ P < 0.01, miRNA adenovirus intervention group (n=6) *vs*. IGF-1+scramble group (n=6). Data are presented as means ± SD. Statistical significance was evaluated with the two-tailed Student's t test **(B**, **C**, **E)** and one-way analysis of variance with Bonferroni *post-hoc* analysis **(G**–**J**, **L**–**N)**.

H9C2 cells were incubated with IGF-1 (10 μM) for 48 h to induce cardiomyocyte hypertrophy. As shown in **Figures 4A, B**, the cells stimulated with IGF-1 were markedly hypertrophic. However, IGF-1 upregulated the mRNA level of ANP and BNP (P < 0.05), but not α-actin (**Figure 4C**) (P > 0.05). Meanwhile, IGF-1 promoted autophagy activity with increased LC3II and Beclin1, and decreased SQSTM1 protein levels (P < 0.01, respectively) (**Figures 4D, E**). Then, the IGF-1 treated H9C2 cells were subjected to adenovirus-mediated miRNA infection. The cell surface area showed a significant decrease in the IGF-1+Ad-26b-5p, IGF-1+Ad-204-5p, and IGF-1+Ad-497-3p groups (**Figure 4F, G**) (P < 0.01, P < 0.05, P < 0.01, respectively), but no significant changes were observed in the other groups (P > 0.05). In addition, overexpression of miR-26b-5p, miR-204-5p, and miR-497-3p attenuated IGF-1 induced cardiomyocyte hypertrophy by downregulating ANP and BNP in both mRNA (**Figures 4H, I**) and protein levels (**Figures 4K–M**), while leaving α-actin unchanged (**Figures 4J, K, N**). These data indicated that IGF-1 stimulation obviously induced cardiomyocyte hypertrophy and that miR-26b-5p, miR-204-5p, and miR-497-3p could inhibit it.

**Figure 5:**
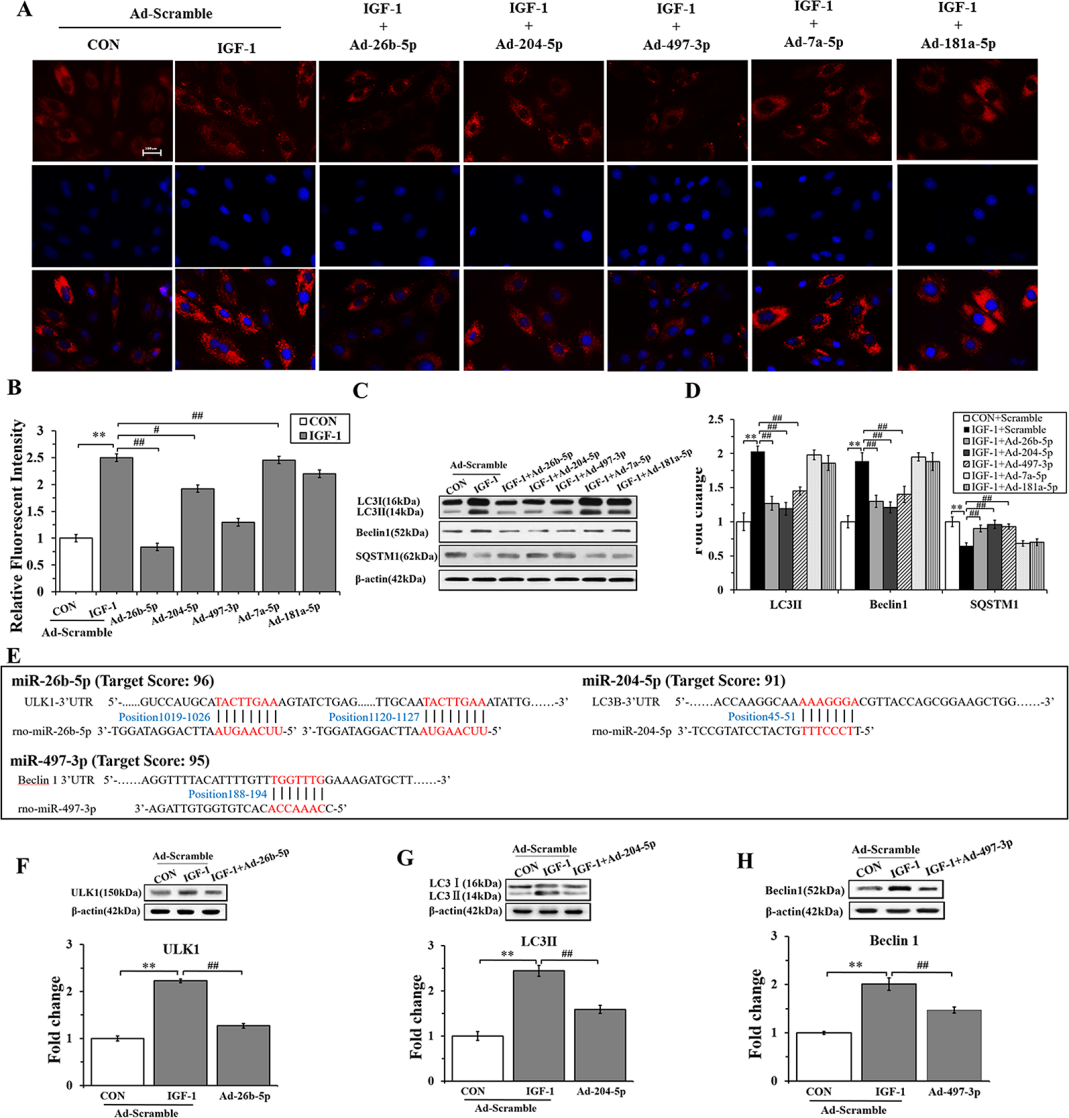
miR-26b-5p, miR-204-5p, and miR-497-3p attenuate IGF-1 induced cardiomyocyte hypertrophy by suppressing autophagy **(A)**, microRNAs (miRNAs) adenovirus intervention in hypertrophic cardiomyocyte, LC3B immunofluorescence staining in various groups. **(B)** The LC3B fluorescent intensity in the various groups. **(C**, **D)**, Western blot analysis showing the expression of the autophagy markers (LC3B, Beclin 1, and SQSTM1). **(E)** The binding sites of miR-26b-5p, miR-204-5p, and miR-497-3p predicted using TargetScan. **(F)** Expression of LC3B protein in H9C2s and H9C2s subjected to adenovirus-mediated miR-204-5p over-expression or the control adenovirus. **(G)** Expression of ULK1 protein in H9C2 cells and in H9C2 cells subjected to adenovirus-mediated miR-26b-5p over-expression or to control adenovirus. **(H)** Expression of Beclin 1 protein in H9C2 cells and in H9C2 cells subjected to adenovirus-mediated miR-497-3p over-expression or control adenovirus. ** P < 0.01, IGF+scramble (n=6) *vs*. CON+scramble group (n=6). ^#^P < 0.05, ^##^ P < 0.01, miRNA adenovirus intervention group (n=6) *vs*. IGF-1+scramble group (n=6). Data are presented as means ± SD. Statistical significance was evaluated with one-way analysis of variance with Bonferroni *post-hoc* analysis.

To gain insights into the effects of miRNAs on IGF-1-induced autophagy, we performed immunofluorescence staining and western blot analysis. As shown in **Figures 5A, B**, Ad-26b-5p, Ad-204-5p, and Ad-497-3p infections significantly decreased the IGF-1 induced expression of LC3 II in cardiomyocyte hypertrophy, as reflected by reduced fluorescence (P < 0.01, P < 0.05, P < 0.01, respectively). The results of the western blot assay were consistent with those of immunofluorescence staining (**Figures 5C, D**). The infection with Ad-26b-5p, Ad-204-5p, and Ad-497-3p significantly decreased the IGF-1-induced expression of LC3 II and Beclin 1 proteins in cardiomyocyte hypertrophy (P < 0.01). Moreover, a marked increase in SQSTM1 was also observed (P < 0.01). In contrast, the levels of LC3 II, Beclin 1, and SQSTM1 were not changed significantly in cells infected with Ad-7a-5p and Anti-181a-5p (P > 0.05).

In addition, to investigate the mechanism of miR-26b-5p, miR-204-2p, and miR-497-3p mediated amelioration of cardiomyocyte hypertrophy induced by IGF-1, we predicted that the autophagy-related genes, ULK1, LC3B, and Beclin 1, were the target genes of miR-26b-5p, miR-204-5p, and miR-497-3p, respectively (**Supplementary Table 3**). The predicted binding sites of miR-26b-5p, miR-204-5p, and miR-497-3p in the 3′-UTR of ULK1, LC3B, and Beclin 1, respectively, are listed in **Figure 5E**. The western blot analysis showed that in the cardiomyocytes infected with Ad-26b-5p, Ad-204-5p, and Ad-497-3p, the protein levels of these predicted target genes were markedly decreased (P < 0.01) (**Figures 5F–H**). These data indicated that IGF-1 induced cardiomyocyte hypertrophy was significantly suppressed by miR-26b-5p, miR-204-5p, and miR-497-3p through autophagy inhibition.

## Discussion

The degree of physiologic hypertrophy is associated with the intensity and duration of the exercise training and is also related to the aerobic or anaerobic metabolism (de Bold et al., 2001). Although cardiac hypertrophy induced by treadmills is widely observed, it has failed to induce cardiac hypertrophy in some cases (Wang et al., 2010). Accumulated evidences demonstrated that swimming training leads to LVH (Geenen et al., 1996; Pelliccia and Maron, 1997; Medeiros et al., 2004) and the degree of the hypertrophy is associated with the endurance (Kaplan et al., 1994; Evangelista and Krieger, 2006). Indeed, some researchers have observed that the swimming training induces robust cardiac hypertrophy when compared to treadmill training in rats and mice (Medeiros et al., 2004; Evangelista and Krieger, 2006; Oliveira et al., 2009; Wang et al., 2010; Soci et al., 2011). Thus, we chose the swimming training to study the mechanisms of physiological LVH. To induce physiological LVH in rats, we referred to the Bedford animal exercise load standard for setting the exercise intensity (Bedford et al., 1979). The 60-min swimming exercise employed in the present study was of moderate intensity for the rats. The 10-week moderate-intensity swimming exercise enlarged and thickened cardiomyocytes. Although an increase in cardiomyocyte volume is also observed in pathological cardiomegaly, increased fetal gene expression such as ANP, BNP, β-MHC were also significantly upregulated in pathological LVH (Swynghedauw, 1986; Dorn et al., 1994; Bernardo et al., 2010; Nakamura and Sadoshima, 2018; Oldfield et al., 2019). In our swimming exercise-induced cardiomyocyte hypertrophy, the expression of ANP, α-actin, and the ratio of β-MHC to α-MHC was not significantly changed, indicating a physiological LVH model was successfully induced. Previous studies have shown that physiological cardiac hypertrophy is associated with an exercise-induced increase in the levels of IGF-1 (Neri Serneri et al., 2001). We found that the cardiomyocyte surface area in response to IGF-1 for 48 hours was significantly larger than that of the non-stimulated H9C2 cells, and the levels of hypertrophy markers (ANP and BNP) were also significantly increased in mRNA levels, while leaving α-actin mRNA unchanged, which is considered as a trigger for physiological cardiac hypertrophy (McMullen and Izumo, 2006; Weeks et al., 2017).

Autophagy is a highly conserved and ubiquitous metabolic pathway in living organisms (Sandoval et al., 2008). Previous studies have demonstrated that Beclin 1 is the earliest self-localizing gene in the structure of autophagic precursors and is believed to regulate other autophagic genes (Maejima et al., 2016; Sun et al., 2018), cardiac-specific overexpression of Beclin-1 promoted autophagy, and improved cardiac function. We observed a dramatic increase in LC3II and Beclin 1 in physiological LVH of rat. In addition, 5 weeks of exercise induced activation of the autophagic pathway including enhanced expression of Atg5 and Atg7 and increased the LC3-II/LC3-I ratio (Willis et al., 2013). Knocking-out of the Atg5 gene or silencing of the Atg7 gene in cardiomyocytes, decreased the autophagic activity (Bernardo et al., 2010). We then measured the expression of Atg5 and Atg7, and found that the two genes were also obviously upregulated in LVH. Moreover, we found that the expression of other key autophagy-related genes such as ULK1, Atg12, and Atg4B, was significantly upregulated at the protein level in the left ventricle of rats with exercise-induced myocardial hypertrophy, whereas SQSTM1 (a marker of reduced autophagy) was downregulated. The findings are in concordance with previous results demonstrating that autophagy is enhanced by exercise training (He et al., 2012; Yan et al., 2017; Li et al., 2019; Moradi et al., 2019), and endurance exercise provided cardioprotective effect by upregulating autophagy (Lee et al., 2017). Consistently, a highly activated autophagy was also observed in IGF-1 induced cardiomyocyte hypertrophy, which was agreed with that IGF-1 inhibition attenuated autophagosome formation (Renna et al., 2013). Taken together, these results suggest that exercise training can increase the autophagy in cardiomyocytes, which plays an important role in physiological cardiac hypertrophy.

Exercise can induce cardiomegaly by upregulating or downregulating certain miRNAs in the myocardium and regulates cardiac hypertrophy and proliferation, cardiovascular regeneration, and myocardial interstitial hypertrophy (Fernandes et al., 2015; Ramasamy et al., 2015). A number of studies have shown that miRNAs play a key role in the regulation of autophagic pathways in different tissues (Frost and Olson, 2011; Lee et al., 2011; Lee et al., 2012; Patnaik et al., 2012; Roggli et al., 2012). Based on bioinformatics analysis, we found the target genes of the differential miRNAs between normal and physiological LVH were enriched in autophagy process. Three highly homologous miRNAs, miR-26b-5p, miR-204-5p, and miR-497-3p, were markedly decreased in rat heart with physiological LVH, which was consistent with the enhanced autophagy observed in physiological LVH. Moreover, overexpression of miR-26b-5p, miR-204-5p, and miR-497-3p attenuated IGF-1 induced cardiomyocyte hypertrophy by downregulating autophagy related genes. In agreement with our results, cardiac and plasma miR-26b expressions were significantly reduced in the transverse aortic constriction (TAC) induced cardiac hypertrophy model of rats (Zhang et al., 2013.). Overexpression of miR-26b reduced TAC- induced cardiac hypertrophy in mice (Han et al., 2012). In ischemia-reperfusion induced cardiomyocytes autophagy, miR-204 targeted LC3-II protein and was significantly down-regulated (Xiao et al., 2011). MiR-497 was also significantly reduced in Ang II-induced cardiomyocytes and TAC mice, overexpression of miR-497 reversed Ang II-induced cardiomyocytes protein synthesis and suppressed cardiac hypertrophy in TAC mice (Xiao et al., 2016). Thus, decreased miR-26b, miR-204 and miR-497 were associated with the increased autophagy and cardiac hypertrophy. Previous studies also demonstrated that ULK1 and LC3B were activated during cardiac hypertrophy, and Beclin1 mediated autophagy was also enhanced during right ventricular remodeling (Huang et al., 2015; Deng et al., 2017; Zhang et al., 2019), supporting our finding that ULK1, LC3B, and Beclin1 were up-regulated in physiological LVH. Given LVH in response to endurance exercise is protective, the miRNA changes in physiological LVH is supposed to be adaptive and to promote moderate autophagy which is essential for physiological LVH. Of note, miR-26b-5p, miR-204-5p, and miR-497-3p had been detected in plasma and were used as a potential diagnostic biomarker for diseases such as lung cancer (Du et al., 2015; Guo et al., 2015; Lu et al., 2018; Nakata et al., 2019). Distinct plasma gradients of miR-204-5p in the pulmonary circulation were observed in patients with different pulmonary hypertension subtypes (Estephan et al., 2019). Thus, the dynamic profile of these miRNA in plasma may help distinguish physiological and pathological LVH.

Taken together, cardiomyocyte autophagy has been considered to play a key role in controlling the hypertrophic response; miR-26b-5p, miR-204-5p, and miR-497-3p were found to play a major role in physiological cardiac hypertrophy by targeting their respective autophagy genes.

## Conclusions

Our results demonstrate that long-term endurance swimming exercise may induce physiological LVH, at least in part, by modulating the microRNA–autophagy axis.

## Data Availability Statement

Our microarray data submitted to GEO has been assigned the GEO accession number GSE143935.

## Ethics Statement

All researches involving animals were conducted according to animal ethics guidelines and approved by the Animal Care and Use Committee of Shanghai Normal University (Shanghai, China).

## Author Contributions

JZ designed the project and JQ contributed to data analysis and wrote the paper. XL developed the experimental design and contributed to data generation. BZ conducted the mRNA analysis from cardiac tissue. ZM and SL analyzed the protein expression in cardiac tissue and contributed to paper writing. JQ, XL, ZM, and BZ contributed to the work equally. All authors read and approved the manuscript.

## Funding

This study was supported financially by the National Natural Science Foundation of China (Grant no. 31571223).

## Conflict of Interest

The authors declare that the research was conducted in the absence of any commercial or financial relationships that could be construed as a potential conflict of interest.

## References

[B1] Aredia F. Scovassi A. I. (2017). A new function for miRNAs as regulators of autophagy. Future Med. Chem. 9 (1), 25–36. 10.4155/fmc-2016-0173 27957876

[B2] Bedford T. G. Tipton C. M. Wilson N. C. Oppliger R. A. Gisolfi C. V. (1979). Maximum oxygen consumption of rats and its changes with various experimental procedures. J. Appl. Physiol. Respir. Environ. Exercise Physiol. 47 (6), 1278–1283. 10.1152/jappl.1979.47.6.1278 536299

[B3] Bernardo B. C. Weeks K. L. Pretorius L. McMullen J. R. (2010). Molecular distinction between physiological and pathological cardiiac hypertrophy: Experimental findings and therapeutic strategies. Pharmacol. Ther. 128 (1), 191–227. 10.1016/j.pharmthera.2010.04.005 20438756

[B4] Bernardo B. C. Ooi J. Y. Y. Weeks K. L. Patterson N. L. McMullen J. R. (2018). Understanding key mechanisms of exercise-induced cardiac protection to mitigate disease: current knowledge and emerging concepts. Physiol. Rev. 98 (1), 419–475. 10.1152/physrev.00043.2016 29351515

[B5] Brandt N. Gunnarsson T. P. Bangsbo J. Pilegaard H. (2018). Exercise and exercise training-induced increase in autophagy markers in human skeletal muscle. Physiol. Rep. 6 (7), e13651. 10.14814/phy2.13651 29626392 PMC5889490

[B6] Carè A. Catalucci D. Felicetti F. Bonci D. Addario A. Gallo P. (2007). MicroRNA-133 controls cardiac hypertrophy. Nat. Med. 13 (5), 613–618. 10.1038/nm1582 17468766

[B7] Chen L. Zhou Y. Sun Q. Zhou J. Pan H. Sui X. (2017). Regulation of Autophagy by MiRNAs and their emerging roles in tumorigenesis and cancer treatment. Int. Rev. Cell Mol. Biol. 334, 1–26. 10.1016/bs.ircmb.2017.03.003 28838537

[B8] de Bold A. J. Ma K. K. Zhang Y. de Bold M. L. Bensimon M. Khoshbaten A. (2001). The physiological and pathophysiological modulation of the endocrine function of the heart. Can. J. Physiol. Pharmacol. 79 (8), 705–714. 10.1139/cjpp-79-8-705 11558679

[B9] Deng Y. Wu W. Guo S. Chen Y. Liu C. Gao X. (2017). Altered mTOR and Beclin-1 mediated autophagic activation during right ventricular remodeling in monocrotaline-induced pulmonary hypertension. Respir. Res. 18 (1), 53. 10.1186/s12931-017-0536-7 28340591 PMC5366117

[B10] Dorn G. W. 2nd Robbins J. Ball N. Walsh R. A. (1994). Myosin heavy chain regulation and myocyte contractile depression after LV hypertrophy in aortic-banded mice. Am. J. Physiol. 267 (1 Pt 2), H400–H405. 10.1152/ajpheart.1994.267.1.H400 8048605

[B11] Dorn G. W. 2nd (2007). The fuzzy logic of physiological cardiac hypertrophy. Hypertension 49 (5), 962–9705. 10.1161/HYPERTENSIONAHA.106.079426 17389260

[B12] Du M. L. Shi D. N. Yuan L. Li P. C. Chu H. Y. Qin C. (2015). Circulating miR-497 and miR-663b in plasma are potential novel biomarkers for bladder cancer. Sci. Rep. 5, 10437. 10.1038/srep10437 26014226 PMC4444850

[B13] Estephan L. E. Genuardi M. V. Kosanovich C. M. Risbano M. G. Zhang Y. Petro N. (2019). Distinct plasma gradients of microRNA-204 in the pulmonary circulation of patients suffering from WHO Groups I and II pulmonary hypertension. Pulm. Circ. 9 (2), 2045894019840646. 10.1177/2045894019840646 PMC6440051 30854934

[B14] Evangelista F. S. Krieger J. E. (2006). Small gene effect and exercise training-induced cardiac hypertrophy in mice: an Ace gene dosage study. Physiol. Genomics 27 (3), 231–236. 10.1152/physiolgenomics.00022.2006 16926272

[B15] Fernandes T. Hashimoto N. Y. Magalha˜es F. C. Fernandes F. B. Casarini D. E. Carmona A. K. (2011). Aerobic exercise training-induced left ventricular hypertrophy involves regulatory microRNAs, decreased angiotensin-converting enzyme-angiotensin II, and synergistic regulation of angiotensin converting enzyme 2-angiotensin (1–7). Hypertension 58 (2), 182–1895. 10.1161/HYPERTENSIONAHA.110.168252 21709209 PMC3184458

[B16] Fernandes T. Baraúna V. G. Negrão C. E. Phillips M. I. Oliveira E. M. (2015). Aerobic exercise training promotes physiological cardiac remodeling involving a set of microRNAs. Am. J. Physiol. Heart Circ. Physiol. 309 (4), H543–H552. 10.1152/ajpheart.00899.2014 26071549 PMC4537939

[B17] Fritzen A. M. Madsen A. B. Kleinert M. Treebak J. T. Lundsgaard A. M. Jensen T. E. (2016). Regulation of autophagy in human skeletal muscle: effects of exercise, exercise training and insulin stimulation. J. Physiol. 594 (3), 745–761. 10.1113/JP271405 26614120 PMC5341711

[B18] Frost R. J. Olson E. N. (2011). Control of glucose homeostasis and insulin sensitivity by the Let-7 family of microRNAs. Proc. Natl. Acad. Sci. U. S. A. 108 (52), 21075–21080. 10.1073/pnas.1118922109 22160727 PMC3248488

[B19] Geenen D. L. Malhotra A. Buttrick P. M. (1996). Angiotensin receptor 1 blockade does not prevent physiological cardiac hypertrophy in the adult rat. J. Appl. Physiol. 81 (2), 816–821. 10.1152/jappl.1996.81.2.816 8872651

[B20] Gottlieb R. A. Mentzer R. M. Jr. (2013). Autophagy: an affair of the heart. Heart Fail Rev. 18 (5), 575–584. 10.1007/s10741-012-9367-2 23188163 PMC3782636

[B21] Guo W. Zhang Y. Zhang Y. Shi Y. Xi J. Fan H. (2015). Decreased expression of miR-204 in plasma is associated with a poor prognosis in patients with non-small cell lung cancer. Int. J. Mol. Med. 36 (6), 1720–1726. 10.3892/ijmm.2015.2388 26497897

[B22] Halling J. F. Pilegaard H. (2017). Autophagy-dependent beneficial effects of exercise. Cold Spring Harb Perspect. Med. 7 (8), a029777. 10.1101/cshperspect.a029777 28270532 PMC5538402

[B23] Han M. Yang Z. Sayed D. He M. Gao S. Lin L. (2012). GATA4 expression is primarily regulated *via a* miR-26b-dependent post-transcriptional mechanism during cardiac hypertrophy. Cardiovasc. Res. 93 (4), 645–654. 10.1093/cvr/cvs001 22219180 PMC3291090

[B24] He C. Bassik M. C. Moresi V. Sun K. Wei Y. Zou Z. (2012). Exercise-induced Bcl_2_-regulated autophagy is required for muscle glucose homeostasis. Nature 481 (7382), 511–515. 10.1038/nature10758 22258505 PMC3518436

[B25] Huang J. Pan W. Ou D. Dai W. Lin Y. Chen Y. (2015). LC3B, a Protein that serves as an autophagic marker, modulates Angiotensin II-induced Myocardial Hypertrophy. J. Cardiovasc. Pharmacol. 66 (6), 576–583. 10.1097/FJC.0000000000000306 26284810

[B26] Jacobsen A. Silber J. Harinath G. Huse J. T. Schultz C. (2013). Analysis of microRNA-target interactions across diverse cancer types. Nat. Struct. Mol. Biol. 20, 1325–1332. 10.1038/nsmb.2678 24096364 PMC3982325

[B27] Kaplan M. L. Cheslow Y. Vikstrom K. Malhotra A. Geenen D. L. Nakouzi A. (1994). Cardiac adaptations to chronic exercise in mice. Am. J. Physiol. 267 (3 Pt 2), H1167–H1173. 10.1152/ajpheart.1994.267.3.H1167 8092282

[B28] Lee E. K. Lee M. J. Abdelmohsen K. Kim W. Kim M. M. Srikantan S. (2011). miR-130 suppresses adipogenesis by inhibiting peroxisome proliferator- activated receptor gamma expression. Mol. Cell Biol. 31 (4), 626–638. 10.1128/MCB.00894-10 21135128 PMC3028659

[B29] Lee S. T. Chu K. Jung K. H. Kim J. H. Huh J. Y. Yoon H. (2012). miR-206 regulates brain derived neurotrophic factor in Alzheimer disease model. Ann. Neurol. 72 (2), 269–277. 10.1002/ana.23588 22926857

[B30] Lee Y. Kwon I. Jang Y. Song W. Cosio-Lima L. M. Roltsch M. H. (2017). Potential signaling pathways of acute endurance exercise-induced cardiac autophagy and mitophagy and its possible role in cardioprotection. J. Physiol. Sci. 67 (6), 639–654. 10.1007/s12576-017-0555-7 28685325 PMC5684252

[B31] Li Y. Yao M. Zhou Q. Cheng Y. Che L. Xu J. (2018b). Dynamic regulation of circulating microRNAs during acute exercise and long-term exercise training in basketball athletes. Front. Physiol. 9, 282. 10.3389/fphys.2018.00282 29662456 PMC5890107

[B32] Li J. Y. Pan S. S. Wang J. Y. Lu J. (2019). Changes in autophagy levels in rat myocardium during exercise preconditioning-initiated cardioprotective effects. Int. Heart J. 60 (2), 419–428. 10.1536/ihj.18-310 30745541

[B33] Lira V. A. Okutsu M. Zhang M. Greene N. P. Laker R. C. Breen D. S. (2013). Autophagy is required for exercise training-induced skeletal muscle adaption and improvement of physical performance. FASEB J. 27 (10), 4184–4193. 10.1096/fj.13-228486 23825228 PMC4046188

[B34] Liu W. Wang X. (2019). Prediction of functional microRNA targets by integrative modeling of microRNA binding and target expression data. Genome Biol. 20 (1), 18. 10.1186/s13059-019-1629-z 30670076 PMC6341724

[B35] Lowes B. D. Minobe W. Abraham W. T. Rizeq M. N. Bohlmeyer T. J. Quaife R. A. (1997). Changes in gene expression in the intact human heart. J. Clin. Invest. 100 (9), 315–2324. 10.1172/JCI119770 PMC508428 9410910

[B36] Lu S. Kong H. Hou Y. Ge D. Huang W. Ou J. (2018). Two plasma microRNA panels for diagnosis and subtype discrimination of lung cancer. Lung Cancer 123, 44–51. 10.1016/j.lungcan.2018.06.027 30089594

[B37] Ma Z. C. Qi J. Meng S. Wen B. J. Zhang J. (2013). Swimming exercise training-induced left ventricular hypertrophy involves microRNAs and synergistic regulation of the PI3K/AKT/mTOR signaling pathway. Eur. J. Appl. Physio.l. 113 (10), 2473–2486. 10.1007/s00421-013-2685-9 23812090

[B38] Maejima Y. Isobe M. Sadoshima J. J. (2016). Regulation of autophagy by Beclin 1 in the heart. Mol. Cell Cardiol. 95, 19–25. 10.1016/j.yjmcc.2015.10.032 PMC4861696 26546165

[B39] Martinelli N. C. Cohen C. R. Santos K. G. Castro M. A. Biolo A. Frick L. (2014). An analysis of the global expression of microRNAs in an experimental model of physiological left ventricular hypertrophy. PloS One 9 (4), e93271. 10.1371/journal.pone.0093271 24751578 PMC3994002

[B40] Martin-Rincon M. Morales-Alamo D. Calbet J. A. L. (2018). Exercise-mediated modulation of autophagy in skeletal muscle. Scand. J. Med. Sci. Sports 28 (3), 772–781. 10.1111/sms.12945 28685860

[B41] McMullen J. R. Izumo S. (2006). Role of the insulin-like growth factor 1 IGF1)/phosphoinositide- 3 - kinase (PI3K) pathway mediating physiological cardiac hypertrophy. Novartis Found. Symp. 274, 90–111. discussion 111-117, 152-155, 272-276. 10.1002/0470029331.ch7 17019808

[B42] Medeiros A. Oliveira E. M. Gianolla R. Casarini D. E. Negrao C. E. Brum P. C. (2004). Swimming training increases cardiac vagal activity and induces cardiac hypertrophy in rats. Braz. J. Med. Biol. Res. 37 (12), 1909–1917. 10.1590/s0100-879x2004001200018 15558199

[B43] Melo S. F. Barauna V. G. Júnior M. A. Bozi L. H. Drummond L. R. Natali A. J. (2015). Resistance training regulates cardiac function through modulation of miRNA-214. Int. J. Mol. Sci. 16 (4), 6855–6867. 10.3390/ijms16046855 25822872 PMC4424992

[B44] Moradi F. Imani A. R. Faghihi M. (2019). Effects of regular exercise plus food restriction on left ventricular pathological remodeling in heart failure-induced rats. Bratisl Lek Listy 120(4), 243–248. 10.4149/BLL_2019_044 31023045

[B45] Nadal M. Gold S. E. (2012). Assessment of autophagosome formation by transmission electron microcopy. Methods Mol. Biol. 835, 481–489. 10.1007/978-1-61779-501-5_29 22183672

[B46] Nakamura M. Sadoshima J. (2018). Mechanisms of physiological and pathological cardiac hypertrophy. Nat. Rev. Cardiol. 15 (7), 387–407. 10.1186/s12917-019-1944-3 29674714

[B47] Nakata K. Heishima K. Sakai H. Yamato O. Furusawa Y. Nishida H. (2019). Plasma microRNA miR-26b as a potential diagnostic biomarker of degenerative myelopathy in Pembroke welsh corgis. BMC Vet. Res. 15 (1), 192. 10.1186/s12917-019-1944-3 31182094 PMC6558770

[B48] Neri Serneri G. G. Boddi M. Modesti P. A. Cecioni I. Coppo M. Padeletti L. (2001). Increased cardiac sympathetic activity and insulin-like growth factor-I formation are associated with physiological hypertrophy in athletes. Circ. Res. 89 (11), 977–982. 10.1161/hh2301.100982 11717153

[B49] Oldfield C. J. Duhamel T. A. Dhalla N. S. (2019). Mechanisms for the transition from physiological to pathological cardiac hypertrophy. Can. J. Physiol. Pharmacol. 10.1139/cjpp-2019-0566 31815523

[B50] Oliveira E. M. Sasaki M. S. Cereˆncio M. Barau´na V. G. Krieger J. E. (2009). Local reninangiotensin system regulates left ventricular hypertrophy induced by swimming training independent of circulating renin: a pharmacological study. J. Renin Angiotensin Aldosterone Syst. 10 (1), 15–23. 10.1177/1470320309102304 19286754

[B51] Patnaik S. K. Dahlgaard J. Mazin W. Kannisto E. Jensen T. Knudsen S. (2012). Expression of microRNAs in the NCI-60 cancer cell-lines. PloS One 7 (11), e49918. 10.1371/journal.pone.0049918 23209617 PMC3509128

[B52] Pelliccia A. Maron B. J. (1997). Outer limits of the athlete's heart, the effect of gender, and relevance to the differential diagnosis with primary cardiac diseases. Cardiol. Clin. 15 (3), 381–396. 10.1016/S0733-8651(05)70347-7 9276164

[B53] Ramasamy S. Velmurugan G. Shanmugha Rajan K. Ramprasath T. Kalpana K. (2015). MiRNAs with apoptosis regulating potential are differentially expressed in chronic exercise-induced physiologically hypertrophied hearts. PloS One 10 (13), e0121401. 10.1371/journal.pone.0121401 25793527 PMC4368613

[B54] Renna M. Bento C. F. Fleming A. Menzies F. M. Siddiqi F. H. Ravikumar B. (2013). IGF-1 receptor antagonism inhibits autophagy. Hum. Mol. Genet. 22 (22), 4528–4544. 10.1093/hmg/ddt300 23804751 PMC3889807

[B55] Roggli E. Gattesco S. Caille D. Briet C. Boitard C. Meda P. (2012). Changes in microRNA expression contribute to pancreatic beta-cell dysfunction in prediabetic NOD mice. Diabetes 61 (7), 1742–1751. 10.2337/db11-1086 22537941 PMC3379668

[B56] Sanchez A. M. (2016). Autophagy regulation in human skeletal muscle during exercise. J. Physiol. 594 (18), 5053–5054. 10.1113/JP272993 27629080 PMC5023693

[B57] Sandoval H. Thiagarajan P. Dasgupta S. K. Schumacher A. Prchal J. T. Chen M. (2008). Essential role for Nix in autophagic maturation of erythroid cells. Nature 454 (7201), 232–235. 10.1038/nature07006 18454133 PMC2570948

[B58] Schiattarella G. G. Hill J. A. (2015). Inhibition of hypertrophy is a good therapeutic strategy in ventricular pressure overload. Circulation 131 (16), 1435–1447. 10.1161/CIRCULATIONAHA.115.013894 25901069 PMC4408778

[B59] Shen G. Ren H. Qiu T. Liang D. Xie B. Zhang Z. (2016). Implications of the interaction between miRNAs and Autophagy in Osteoporosis. Calcif. Tissue Int. 99 (1), 1–12. 10.1007/s00223-016-0122-x 26922423

[B60] Soci U. P. Fernandes T. Hashimoto N. Y. Mota G. F. Amadeu M. A. Rosa K. T. (2011). MicroRNAs 29 are involved in the improvement of ventricular compliance promoted by aerobic exercise training in rats. Physiol. Genomics 43 (11), 665–673. 10.1152/physiolgenomics.00145.2010 21447748 PMC3121159

[B61] Sun Y. Yao X. Zhang Q. J. Zhu M. Liu Z. P. Ci B. (2018). Beclin-1-Dependent Autophagy Protects the Heart During Sepsis. Circulation 138 (20), 2247–2262. 10.1161/CIRCULATIONAHA.117.032821 29853517 PMC6274625

[B62] Swynghedauw B. (1986). Developmental and functional adaptation of contractile proteins in cardiac and skeletal muscles. Physiol. Rev. 66 (3), 710–771. 10.1152/physrev.1986.66.3.710 2942954

[B63] Wang Y. Wisloff U. Kemi O. J. (2010). Animal models in the study of exercise-induced cardiac hypertrophy. Physiol. Res. 59 (5), 633–644. 10.1088/0967-3334/31/1/R01 20406038

[B64] Weeks K. L. Bernardo B. C. Ooi J. Y. Y. Patterson N. L. McMullen J. R. (2017). The IGF1-PI3K-Akt signaling pathway in mediating exercise-induced cardiac hypertrophy and protection. Adv. Exp. Med. Biol. 1000, 187–210. 10.1007/978-981-10-4304-8_12 29098623

[B65] Weinberg E. O. Thienelt C. D. Katz S. E. Bartunek J. Tajima M. Rohrbach S. (1999). Gender differences in molecular remodeling in pressure overload hypertrophy. J. Am. Coll. Cardiol. 34 (1), 264–273. 10.1016/s0735-1097(99)00165-5 10400020

[B66] Willis M. S. Min J. N. Wang S. McDonough H. Lockyer P. Wadosky K. M. (2013). Carboxyl terminus of Hsp70-interacting protein (CHIP) is required to modulate cardiac hypertrophy and attenuate autophagy during exercise. Cell Biochem. Funct. 31 (8), 724–735. 10.1002/cbf.2962 23553918 PMC3770741

[B67] Xiao J. Zhu X. He B. Zhang Y. Kang B. Wang Z. (2011). MiR-204 regulates cardiomyocyte autophagy induced by ischemia-reperfusion through LC3-II. J. BioMed. Sci. 18, 35. 10.1186/1423-0127-18-35 21631941 PMC3127824

[B68] Xiao J. J. Xu T. Z. Li J. Lv D. C. Chen P. Zhou Q. L. (2014). Exercise-induced physiological hypertrophy initiates activation of cardiac progenitor cells. Int. J. Clin. Exp. Pathol. 7 (2), 663–669. 24551287 PMC3925911

[B69] Xiao Y. Zhang X. Fan S. Cui G. Shen Z. (2016). MicroRNA-497 inhibits cardiac Hypertrophy by targeting Sirt4. PloS One 11 (12), e0168078. 10.1371/journal.pone.0168078 27992564 PMC5161464

[B70] Yan Z. Kronemberger A. Blomme J. Call J. A. Caster H. M. Pereira R. O. (2017). Exercise leads to unfavourable cardiac remodelling and enhanced metabolic homeostasis in obese mice with cardiac and skeletal muscle autophagy deficiency. Sci. Rep. 7 (1), 7894. 10.1038/s41598-017-08480-2 28801668 PMC5554260

[B71] Zhang Y. Chen N. (2018). Autophagy is a promoter for aerobic exercise performance during high altitude training. Oxid. Med. Cell Longev. 2018, 3617508. 10.1155/2018/3617508 29849885 PMC5907404

[B72] Zhang Z. H. Li J. Liu B. R. Luo C. F. Dong Q. Zhao L. N. (2013). MicroRNA-26 was decreased in rat cardiac hypertrophy model and may be a promising therapeutic target. J. Cardiovasc. Pharmacol. 62 (3), 312–319. 10.1097/FJC.0b013e31829b82e6 23719092

[B73] Zhang A. Wang M. Zhuo P. (2019). Unc-51 like autophagy activating kinase 1 accelerates angiotensin II-induced cardiac hypertrophy through promoting oxidative stress regulated by Nrf-2/HO-1 pathway. Biochem. Biophys. Res. Commun. 509 (1), 32–39. 10.1016/j.bbrc.2018.11.190 30581007

