# Supplementary material for: Downregulation of miR-26b-5p, miR-204-5p, and miR-497-3p Expression Facilitates Exercise-Induced Physiological Cardiac Hypertrophy by Augmenting Autophagy in Rats
Source: Front Genet. 2020 Feb 19;11:78. doi: 10.3389/fgene.2020.00078 (PMC7042403; doi:10.3389/fgene.2020.00078)
Supplement: Supplementary file 1 [file Table_1.docx]

**Supplementary Table 1 Quantitative real-time PCR primer**

| Genes | Sequences |
| --- | --- |
| ANP | F: 5’- CTTCGGGGGTAGGATTGAC -3’ |
|  | R: 5’- CTTGGGATCTTTTGCGAT CT -3’ |
| α-actin | F: 5’- ACCACAGGCATTGTTCTGGA -3’ |
|  | R: 5’- TAAGGTAGTCAGTGAGGTCC -3’ |
| α-MHC | F: 5’- TCATCCACGGCCTCTTG -3’ |
|  | R: 5’- CACCAGAATGGCTCAAG -3’ |
| β-MHC | F: 5’- CGCCTGTCAGCTTGTAAATG -3’ |
|  | R: 5’- ACAACCCCTACGATTATGCG -3’ |
| SERCA-2α | F: 5’- ACATCACACAGTGAGCTGGG -3’ |
|  | R: 5’- ATGTAAGGGTGTTCGGGTGC -3’ |
| LC3B | F: 5’- CATGAGCGAGTTGGTCAAGA -3’ |
|  | R: 5’- TTGACTCAGAAGCCGAAGGT -3’ |
| beclin1 | F: 5’- GGCCAATAAGATGGGTCTGA -3’ |
|  | R: 5’- GCTGCACACAGTCCAGAAAA -3’ |
| GAPDH | F: 5’- ACCACAGTCCATGCCATCAC -3’ |
|  | R: 5’- TCCACCACCCTGTTGCTGTA -3’ |

*^Genes: ANP atrial natriuretic polypeptide, α-actin skeletal muscle α-actin, α-MHC α-myosin heavy chain, β-MHC β-myosin heavy chain, SERCA-2α sarco endoplasmic reticulum Ca2+-ATPase, LC3B Microtubule associated light chain protein 3B^*
