# Supplementary material for: Downregulation of miR-26b-5p, miR-204-5p, and miR-497-3p Expression Facilitates Exercise-Induced Physiological Cardiac Hypertrophy by Augmenting Autophagy in Rats
Source: Front Genet. 2020 Feb 19;11:78. doi: 10.3389/fgene.2020.00078 (PMC7042403; doi:10.3389/fgene.2020.00078)
Supplement: Supplementary file 2 [file Table_2.docx]

**Supplementary Table 2 The DE miRNAs of the pathway in left ventricular**

| **Pathway** | **P-value** | **Selection Counts** | **Enrichment Score** | **miRNAs** |
| --- | --- | --- | --- | --- |
| MAPK signaling pathway - Rattus norvegicus (rat) | 0.000 | 35 | 4.693422 | rno-miR-27a-5p//rno-miR-26b-5p//rno-miR-129-5p//rno-miR-497-3p//rno-miR-488-3p// rno-miR-204-5p//rno-let-7a-5p//rno-miR-466b-4-3p//rno-miR-27b-3p//rno-miR-181a-5p//rno-miR-466b-2-3p//rno-let-7e-5p//rno-miR-195-5p//rno-miR-145-3p//rno-miR-24-1-5p//rno-miR-499-3p |
| Autophagy - animal - Rattus norvegicus (rat) | 0.000 | 18 | 3.904886 | rno-let-7d-5p//rno-miR-93-5p//rno-miR-106b-5p//rno-miR-130-5p//rno-miR-181a-5p//rno-miR-145b-3p//rno-miR-27a-5p//rno-miR-135a-5p//rrno-miR-27b-3p//rno-miR-129-5p//rno-let-7a-5p//rno-miR-204-5p//rno-miR-24-1-5p//rno-miR-206-5p//rno-miR-26b-5p//rno-miR-497-3p |
| Axon guidance - Rattus norvegicus (rat) | 0.000 | 23 | 3.809652 | rno-miR-27a-5p//rno-miR-26b-5p//rno-miR-129-5p//rno-miR-497-3p//rno-miR-488-3p// rno-miR-204-5p//rno-let-7a-5p//rno-miR-24-1-5p//rno-miR-135a-5p |
| Neurotrophin signaling pathway - Rattus norvegicus (rat) | 0.000 | 18 | 3.382826 | rno-miR-27a-5p//rno-miR-26b-5p//rno-miR-129-5p//rno-miR-497-3p//rno-miR-488-3p// rno-miR-204-5p//rno-let-7a-5p//rno-let-7e-5p |
| cAMP signaling pathway - Rattus norvegicus (rat) | 0.001 | 23 | 3.286073 | rno-miR-27a-5p//rno-miR-26b-5p//rno-miR-129-5p//rno-miR-497-3p//rno-miR-488-3p// rno-miR-204-5p//rno-let-7a-5p//rno-miR-145-3p |
| Lysosome - Rattus norvegicus (rat) | 0.001 | 17 | 3.129122 | rno-miR-27a-5p//rno-miR-26b-5p//rno-miR-129-5p//rno-miR-497-3p//rno-miR-488-3p// rno-miR-204-5p//rno-let-7a-5p/rno-miR-466b-2-3p//rno-miR-499-3p |
| Mitophagy - animal - Rattus norvegicus (rat) | 0.001 | 11 | 3.095096 | rno-miR-488-3p//rno-let-7a-5p//rno-miR-26b-5p//rno-miR-204-5p//rno-miR-206-5p |
| TGF-beta signaling pathway - Rattus norvegicus (rat) | 0.001 | 13 | 3.023684 | rno-miR-27a-5p//rno-miR-26b-5p//rno-miR-129-5p//rno-miR-497-3p//rno-miR-488-3p// rno-miR-204-5p//rno-let-7a-5p//rno-miR-195-5p rno-let-7e-5p |
| Cushing syndrome - Rattus norvegicus (rat) | 0.001 | 19 | 2.96984 | rno-miR-27a-5p//rno-miR-26b-5p//rno-miR-129-5p//rno-miR-497-3p//rno-miR-488-3p// rno-miR-204-5p//rno-let-7a-5p//rno-miR-181a-5p |
| Ras signaling pathway - Rattus norvegicus (rat) | 0.002 | 25 | 2.813991 | rno-miR-27a-5p//rno-miR-26b-5p//rno-miR-129-5p//rno-miR-497-3p//rno-miR-488-3p// rno-miR-204-5p//rno-let-7a-5p//rno-miR-93-5p |
