# Supplementary material for: Downregulation of miR-26b-5p, miR-204-5p, and miR-497-3p Expression Facilitates Exercise-Induced Physiological Cardiac Hypertrophy by Augmenting Autophagy in Rats
Source: Front Genet. 2020 Feb 19;11:78. doi: 10.3389/fgene.2020.00078 (PMC7042403; doi:10.3389/fgene.2020.00078)
Supplement: Supplementary file 3 [file Table_3.docx]

**Supplementary Table 3 The targeted genes of miRNAs**

| **miRNAs** | **Predicted validated target genes**  **(Context score <- 0.2)** | **Autophagy-related target gene** | **MFE** (kcal/mol) | **Seed type** | **Context++ score** |
| --- | --- | --- | --- | --- | --- |
| rno-miR-26b-5p | 456 | ULK1 | -22.3 | Conserved  8mer sites | -0.36 |
| rno-miR-204-5p | 226 | LC3B | -22.6 | Conserved 7mer-m8 sites | -0.20 |
| rno-miR-497-3p | 433 | Beclin1 | -24.5 | Conserved 7mer-m8 sites | -0.23 |
